# Endoscopic hand-suturing for postoperative suture failure

**DOI:** 10.1055/a-2257-3427

**Published:** 2024-02-22

**Authors:** Naosuke Kuraoka, Tetsuro Ujihara, Shun Sakai, Haruhiko Okada, Satoru Hashimoto

**Affiliations:** 1 26379 Department of Gastroenterology, Saiseikai Kawaguchi General Hospital, Kawaguchi, Japan; 2 26379 Department of Gastrointestinal Surgery, Saiseikai Kawaguchi General Hospital, Kawaguchi, Japan

An 82-year-old man underwent robot-assisted total gastrectomy for gastric cancer. On postoperative day 5, a pancreatic leak was observed and open abdominal drainage was performed. Upper gastrointestinal (UGI) endoscopy performed on postoperative day 29 revealed delayed suture failure at the esophagojejunal anastomosis and dissection of the anastomosis ( Fig. 1 ). The patientʼs general condition was poor, and it was difficult to re-suture the anastomosis. The pancreatic leak was managed with drainage, allowing the patient’s nutritional status to improve.

**Fig. 1:**
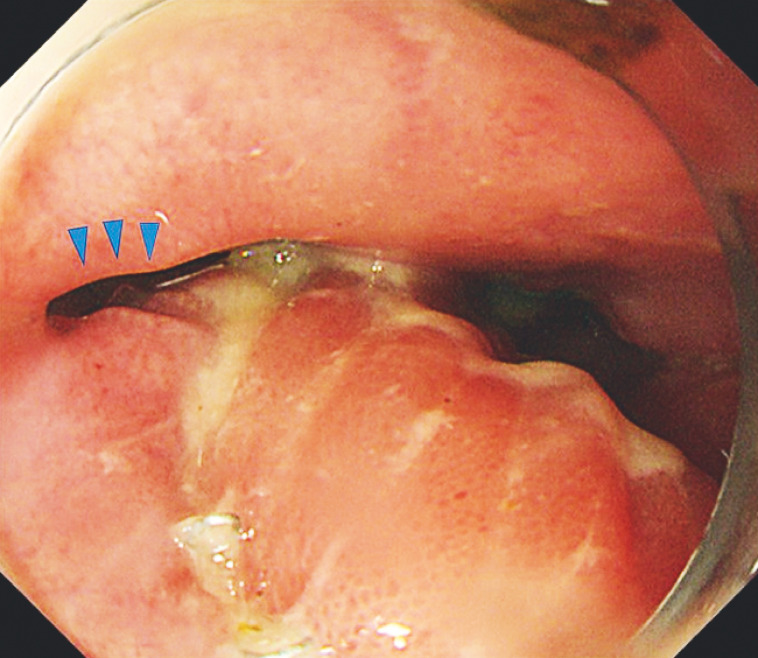
Endoscopic image showing delayed suture failure at the esophagojejunal anastomosis and anastomotic dissection (arrow).

Minimally invasive endoscopic suturing of the esophagojejunal anastomosis was later performed. An endoscopic needle holder (Stuart; Olympus, Tokyo, Japan) and a suture needle with a barbed thread, without the need to tie knots (V-Loc; Covidien, Massachusetts, USA), were used for endoscopic hand-suturing ( Fig. 2 ). The endoscopic hand-suturing closure was performed with continuous sutures from the esophageal side, and the procedure was terminated when the dissected site was closed with the mucosa ( Video 1 ). A UGI series performed 26 days after the endoscopic treatment revealed that the suture failure had resolved, and oral intake was permitted ( Fig. 3 ). A UGI endoscopy 3 months after the endoscopic treatment confirmed that the suture defect had completely closed ( Fig. 4 ).

**Fig. 2:**
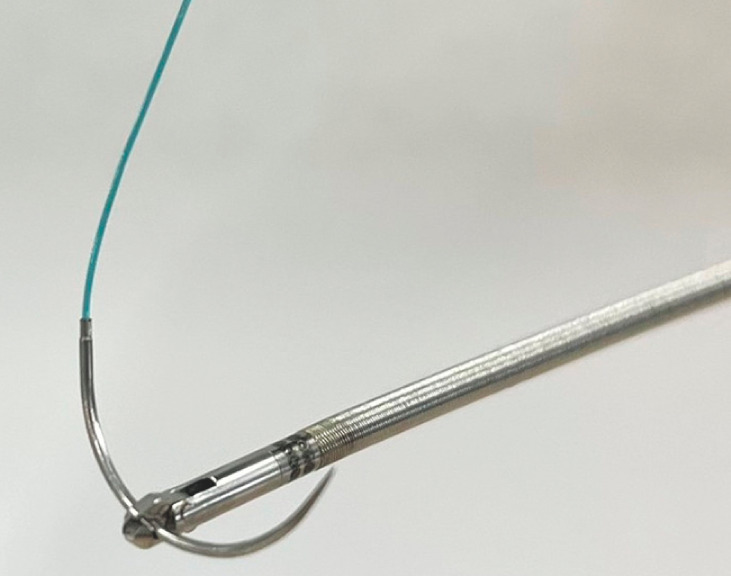
Photograph of the endoscopic needle holder (Stuart, Olympus, Tokyo, Japan) and suture needle with thread (V-Loc, Covidien, Massachusetts, USA).

Endoscopic hand-suturing for postoperative suture failure is performed using a continuous suture.Video 1

**Fig. 3:**
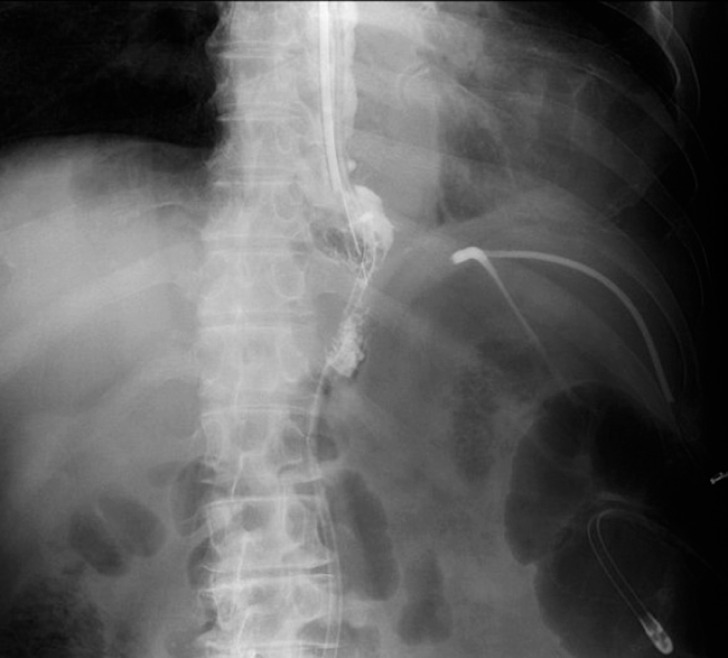
Radiographic image showing resolution of the suture failure.

**Fig. 4:**
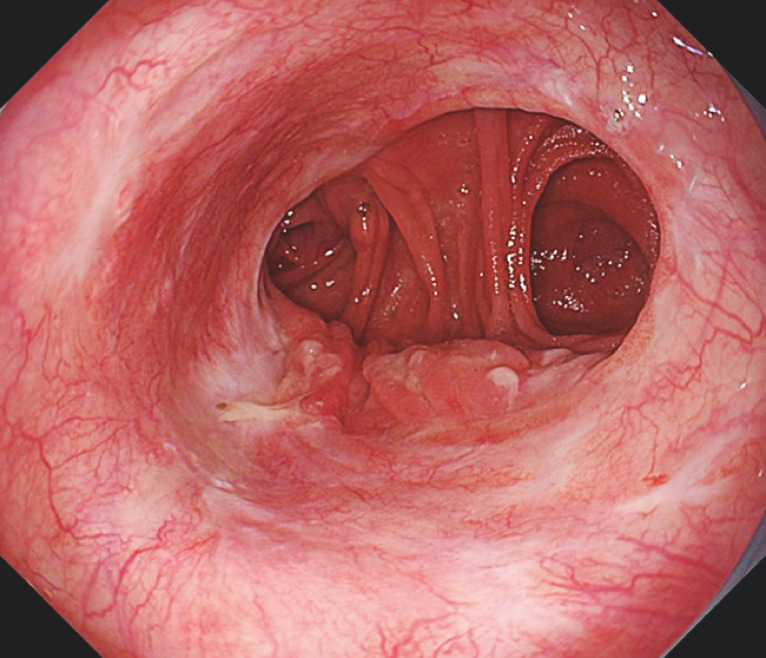
Endoscopic image showing complete closure of the suture defect 3 months after the endoscopic treatment.

The technique of endoscopic hand-suturing using a suture needle with a thread was developed to suture the resection surface during endoscopic submucosal dissection (ESD) for GI tumors 1 2 3 . Recently, a report described the use of this technique for the closure of a fistula after endoscopic ultrasound (EUS)-guided pancreatic cyst drainage 4 . Although there are no reports regarding its use for postoperative suture failure, endoscopic hand-suturing is considered a minimally invasive and effective technique that can be used in patients with poor general condition, for whom there would be difficulties undergoing repeat surgery. Endoscopic hand-suturing has the potential to be used as a minimally invasive endoscopic treatment for postoperative suture failure.

Endoscopy_UCTN_Code_TTT_1AO_2AI

## Notes

Endoscopy E-Videos https://eref.thieme.de/e-videos
*E-Videos* is an open access online section of the journal *Endoscopy* , reporting on interesting cases and new techniques in gastroenterological endoscopy. All papers include a high-quality video and are published with a Creative Commons CC-BY license. Endoscopy E-Videos qualify for HINARI discounts and waivers and eligibility is automatically checked during the submission process. We grant 100% waivers to articles whose corresponding authors are based in Group A countries and 50% waivers to those who are based in Group B countries as classified by Research4Life (see: https://www.research4life.org/access/eligibility/ ). This section has its own submission website at https://mc.manuscriptcentral.com/e-videos .

## References

[1] OSasaki M Akimoto T Endoscopic hand-suturing for defect closure after gastric endoscopic submucosal dissection: a pilot study in animals and in humans Endoscopy 2017 49 792 797 10.1055/s-0043-110668 28561197

[2] Abe S Saito Y Tanaka Y A novel endoscopic hand-suturing technique for defect closure after colorectal endoscopic submucosal dissection: a pilot study Endoscopy 2020 52 780 785 10.1055/a-1120-8533 32207119

[3] Akimoto T Goto O Sasaki M Endoscopic hand suturing for mucosal defect closure after gastric endoscopic submucosal dissection may reduce the risk of postoperative bleeding in patients receiving antithrombotic therapy Dig Endosc 2022 34 123 132 10.1111/den.14045 34021512

[4] Minoda Y Fujimori N Esaki M Rare complications related to lumen-apposing metal stent placement, successfully treated by endoscopic hand-suturing device Endoscopy 2023 55 E692 E693 10.1055/a-2072-5740 37142248 PMC10159776

